# The correlation between intracranial arterial calcification and the outcome of reperfusion therapy

**DOI:** 10.1002/acn3.51780

**Published:** 2023-04-23

**Authors:** Heng Du, Jianrong Zheng, Xuelong Li, Daniel Bos, Wenjie Yang, Yajing Cheng, Cong Liu, Lawrence Ka Sing Wong, Jun Hu, Xiangyan Chen

**Affiliations:** ^1^ Department of Health Technology and Informatics The Hong Kong Polytechnic University Kowloon Hong Kong SAR China; ^2^ Department of Neurology Peking University Shenzhen Hospital Shenzhen China; ^3^ Department of Radiology and Nuclear Medicine, Department of Epidemiology Erasmus MC University Medical Center Rotterdam Netherlands; ^4^ Department of Clinical Epidemiology Harvard TH Chan School of Public Health Boston Massachusetts USA; ^5^ Department of Diagnostic Radiology and Nuclear Medicine University of Maryland School of Medicine Baltimore USA; ^6^ Department of Medicine and Therapeutics, Prince of Wales Hospital The Chinese University of Hong Kong Hong Kong China

## Abstract

**Objective:**

Intracranial arterial calcification (IAC) is a risk factor of ischemic stroke. However, the relationship between IAC patterns and clinical outcome of ischemic stroke remains controversial. We aimed to investigate the correlation between IAC patterns and the effects of reperfusion therapy among acute stroke patients.

**Methods:**

Consecutive acute ischemic stroke patients who underwent reperfusion therapy were included. IAC was categorized as intimal or medial. Based on its involvement, IAC was further classified as diffuse or focal. Neurologic dysfunction was assessed by the National Institute of Health stroke scale (NIHSS). Clinical outcome including favorable neurologic outcome (FNO) and early neurologic deterioration (END) were assessed.

**Results:**

Of 130 patients, 117 had IAC. Intimal IAC was identified in 74.6% of patients and medial IAC was present in 64.6% of patients. Diffuse IAC was present in 31.5% of patients. All diffuse IACs were medial pattern. Diffuse IAC was associated with higher baseline NIHSS (*p* = 0.011) and less FNO (*p* = 0.047). Compared with patients with focal or single diffuse IAC, patients with multiple diffuse IAC had higher baseline NIHSS (*p* = 0.002) and less FNO (*p* = 0.024). Multivariable linear regression (*p* < 0.001) and logistic regression (*p* = 0.027) suggested that multiple diffuse IAC was associated with higher baseline NIHSS and less FNO. No significant association was found between END and different IAC patterns.

**Interpretation:**

Multiple diffuse medial IAC may predict severer neurologic dysfunction and less favorable neurologic outcome after reperfusion therapy in acute stroke patients.

## Introduction

Intracranial arterial calcification (IAC) has been suggested to be an important imaging marker to predict ischemic stroke and guide clinical management of stroke patients.^1^, ^2^ Higher IAC volume was found to be associated with incomplete arterial revascularization after endovascular thrombectomy (EVT)^3^ and hemorrhagic transformation after intravenous thrombolysis (IVT).^4^ Stroke patients with higher IAC burden (number of calcified major intracranial arteries) were reported to experience higher rates of vascular events^5^ and poorer functional outcome.^6^ While most of the previous studies worked on the volume and the burden of IAC, the pattern of IAC has not been fully studied yet. IAC can be classified as intimal and medial pattern based on its location in arterial wall.^7^ Increasing histological and imaging studies suggested that intimal IAC is closely related to atherosclerosis^7^, ^8^ while medial IAC leads to arterial stiffness.^9^ A recent study based on endovascular thrombectomy (EVT) identified that with the presence of medial IAC rather than intimal IAC in the internal carotid artery, patients may benefit in 90‐day functional outcome after EVT, compared with patients who did not undergo EVT.^10^ Accordingly, we hypothesized that among acute stroke patients, IAC patterns are associated with the clinical therapeutic effect on neurologic function recovery in patients who receive reperfusion therapy (IVT and bridging therapy).

In this study, we aimed to investigate the effects of IAC patterns on the outcome of acute stroke patients receiving reperfusion therapy.

## Materials and Methods

### Subjects

The present prospective patient cohort study was approved by the Clinical Research Ethics Committee of the Peking University Shenzhen Hospital. Consecutive patients admitted to the stroke center from 2019 to 2021 were recruited. The inclusion criteria were as follows: (1) patients above 18 years old who were diagnosed as acute cerebral infarction; (2) reperfusion therapies were conducted, including IVT and bridging therapy (IVT with subsequent EVT). IVT was performed within 4.5 h after stroke onset, with or without following endovascular thrombectomy (EVT) within 6 h based on the evaluations of clinical neurologists; (3) non‐contrast brain computed tomography (CT) was performed before reperfusion therapy. Follow‐up brain CT was performed after reperfusion therapy in patients with suspected intracerebral hemorrhage. The exclusion criteria were as follows: (1) contraindications to reperfusion therapy; (2) baseline modified Ranking Scale (mRS) score ≥2; (3) poor quality of imaging data or incomplete clinical data.

### Clinical assessments

Baseline characteristics including gender, age, and vascular risk factors such as hypertension, diabetes, and atrial fibrillation were recorded. The size of ischemic stroke lesion was assessed by the Alberta Stroke Program Early CT score (ASPECTS) in patients with anterior circulation stroke and Posterior Circulation ASPECTS (pc‐ASPECTS) in patients with posterior circulation stroke. The severity of neurological dysfunction was assessed using the National Institute of Health stroke scale (NIHSS). The primary outcome of this study was favorable neurologic outcome (FNO), defined as either NIHSS reduction ≥8 points or final NIHSS ≤3 within 10 days after reperfusion therapy.^11^, ^12^ The secondary outcome was early neurologic deterioration (END), defined as an increase in NIHSS ≥4 points, explainable by stroke compared with the post‐therapy NIHSS, or death within 10 days after reperfusion therapy.^13^ END includes stroke progression, stroke recurrence, symptomatic cerebral hemorrhage, and death.^14^, ^15^ Stroke progression was defined as worsened stroke without intracerebral hemorrhage or stroke recurrence.^13^, ^14^, ^15^ Stroke recurrence was defined as a sudden, persist dysfunction after therapy affecting an artery territory that was different from the original stroke territory.^15^ Symptomatic cerebral hemorrhage was defined as intraparenchymal hemorrhage identified on brain CT.^16^

### Evaluation on intracranial arterial calcification

**Figure 1:**
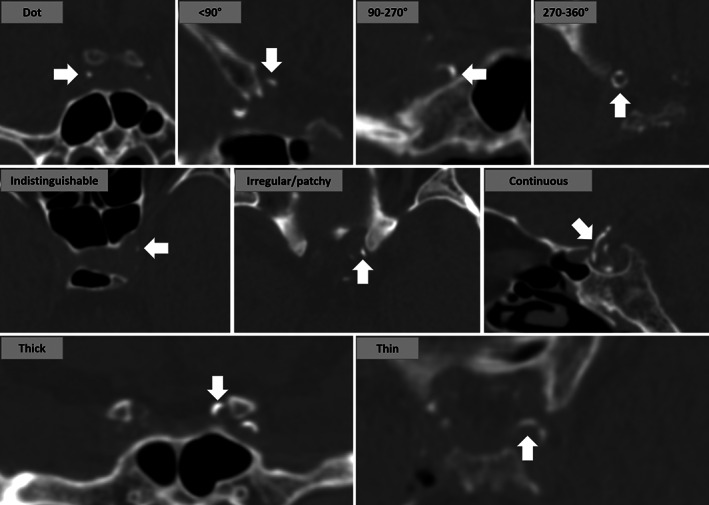
Grading scale of intracranial arterial calcification (IAC). Circularity (1 for dot, 2 for <90°, 3 for 90–270°, and 4 for 270–360°), thickness (1 for thick IAC ≥1.5 mm and 3 for thin IAC <1.5 mm), and morphology (0 for indistinguishable, 1 for irregular/patchy, and 4 for continuous) were assessed.

**Figure 2:**
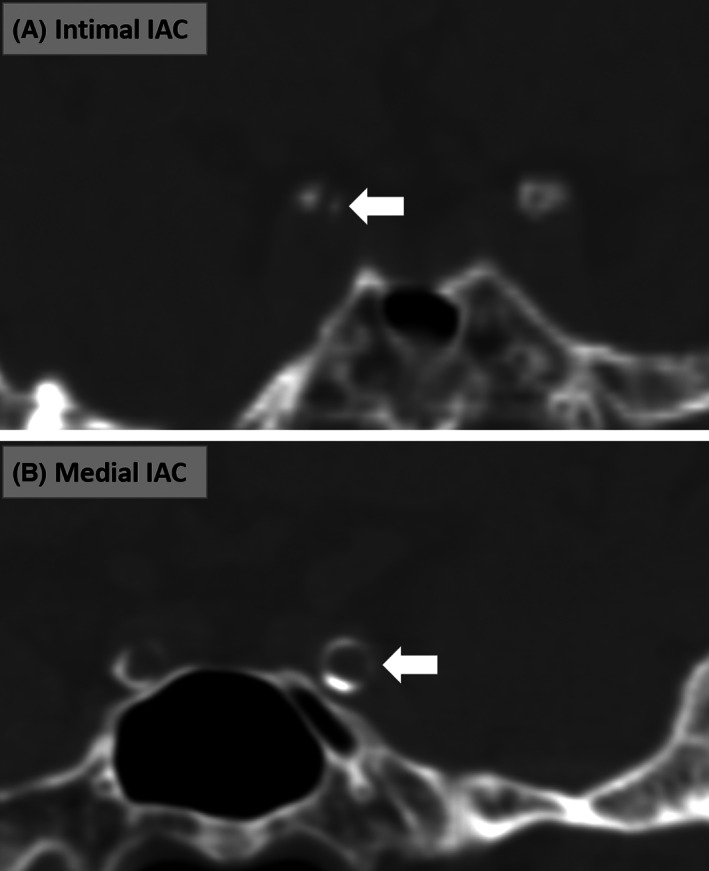
Patterns of intracranial arterial calcification (IAC): intimal IAC (A) and medial IAC (B).

**Figure 3:**
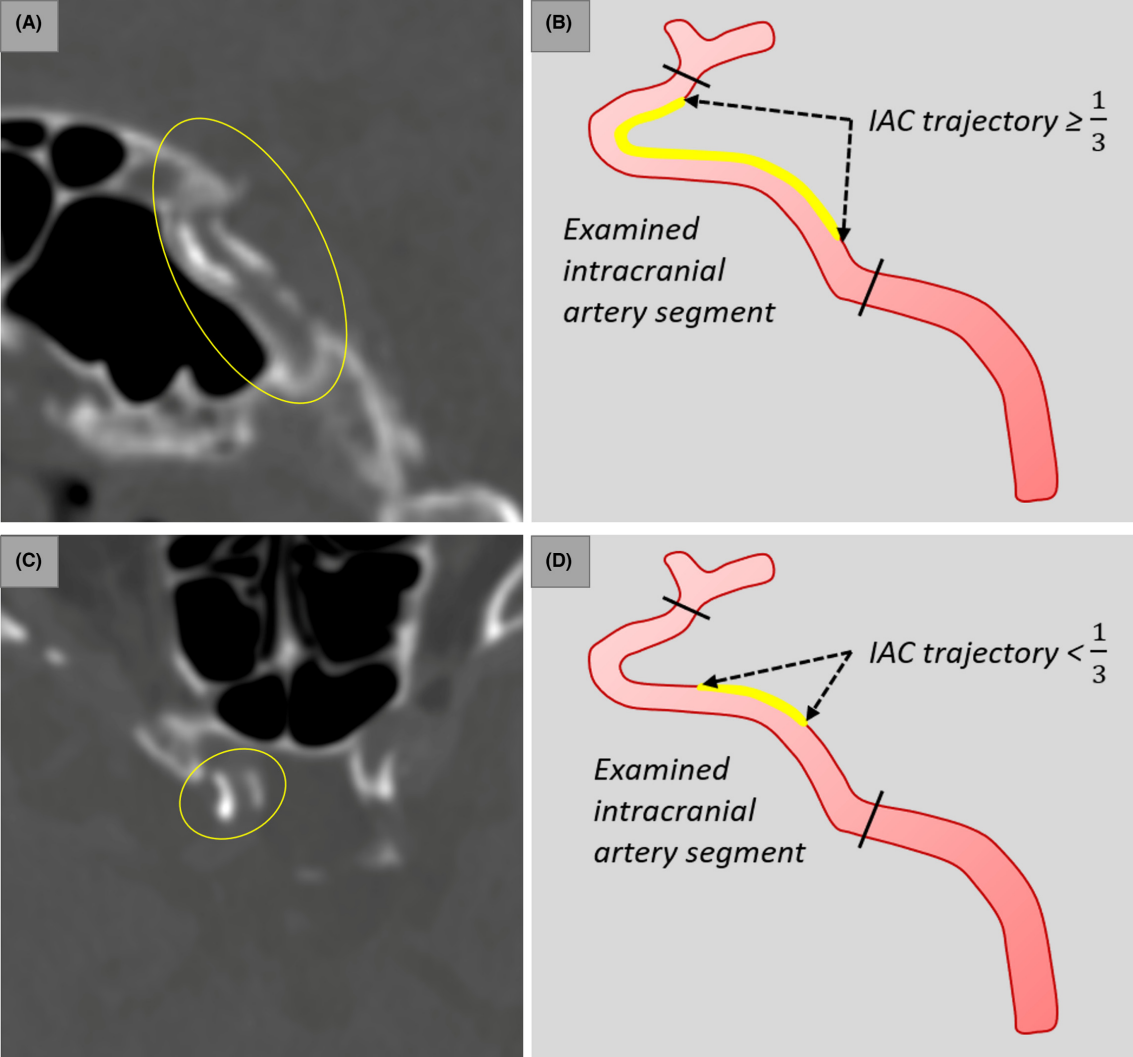
Involvement of intracranial arterial calcification (IAC): diffuse IAC (A) and focal IAC (C). The trajectory of diffuse IAC extended over 1/3 of the length of the examined intracranial artery segment (B) while the trajectory of focal IAC involved less than 1/3 of the examined intracranial artery segment (D).

Intracranial arterial calcification (IAC) was identified on CT images before the procedure of reperfusion therapy. Major intracranial arterial segments were examined, including C3 to C7 segments of the internal carotid artery (ICA), M1 segment of the middle cerebral artery (MCA), V4 segment of the vertebral artery (VA), and the basilar artery (BA). The presence of IAC was defined as hyperdense foci over 130 Hounsfield units. IAC was measured by two independent neurologists (H.D. and X.L.) who were blinded to clinical characteristics. IAC was classified into two pathologic patterns: intimal IAC and medial IAC, based on a previously developed and validated grading scale.^17^ The grading scale assessed circularity (1 point for dot, 2 for <90°, 3 for 90–270°, and 4 for 270–360°), thickness (1 point for thick IAC ≥1.5 mm and 3 for thin IAC <1.5 mm), and morphology (0 point for indistinguishable, 1 for irregular/patchy and 4 for continuous) of each IAC (Fig. 1). A summed score ranging from 1 to 6 indicated predominant intimal IAC (Fig. 2A) and a score from 7 to 11 was considered as medial IAC (Fig. 2B). In patients with IAC, calcification was further categorized based on the involvement, which was defined by the trajectory along intracranial arteries: (1) diffuse IAC was calcification of which the trajectory extended over 1/3 of the examined intracranial arteries (Fig. 3A,B) and (2) focal IAC was defined as the trajectory of IAC involving less than 1/3 of the examined intracranial arteries (Fig. 3C,D). Additionally, single diffuse IAC was defined as only one intracranial artery segment involved by diffuse IAC and multiple diffuse IAC was defined as two or more examined intracranial arteries involved by diffuse IAC.

### Statistical analysis

IBM SPSS (20.0, SPSS, Inc) was used for statistical analysis. Continuous variables were expressed by mean ± standard deviation (SD) and categorical variables were presented as numbers and percentages. The inter‐rater reliability of IAC measurement was assessed by Cohen's kappa analysis. Independent *t*‐test was used for comparisons of age and NIHSS score between different IAC groups (intimal IAC vs. medial IAC; diffuse IAC vs. focal IAC). Pearson's chi‐squared test or Fisher's exact test was used for comparisons of other risk factors, FNO and END between different IAC groups. In the identification of confounding factors, the association between age and FNO or END was based on independent *t*‐tests; the association between other dichotomous confounders and FNO or END was based on Pearson's chi‐square test, Fisher's exact test, or Mann–Whitney *U*‐tests. Factors with p‐value less than 0.1 were considered as confounding factors. Multivariable linear regression was used for examining the correlation between NIHSS and different patterns of IAC. Multivariable binary logistic regression was used to examine the correlation between different patterns of IAC and FNO or END.

## Results

Of the 135 patients that met inclusion criteria, five were excluded because of incomplete scans (*n* = 4) and unavailable clinical data (*n* = 1). Of the 130 patients (mean age ± SD, 64.62 ± 13.69 years old) included in this study, 81 (62.3%) were men. Thirty‐six patients (27.7%) had hypertension, 78 (60.0%) patients had diabetes, 17 (13.1%) patients had hyperlipidemia, and 21 (16.2%) patients had atrial fibrillation. Previous history of stroke or TIA was reported in 43 (33.1%) patients. A history of ischemic heart disease was reported in 23 (17.7%) patients, current smoking in 47 (36.2%) patients, and drinking in 38 (29.2%) patients. Among the 130 stroke patients, 98 (75.4%) had ischemic stroke in the anterior circulation and 32 (24.6%) had ischemic stroke in the posterior circulation. During the treatment procedure (IVT, IVT plus EVT), no patients received general anesthesia or sedation.

Intracranial arterial calcification was identified in 117 (90.0%) patients, with intimal and medial IAC identified in 97 (74.6%) and 84 (64.6%) patients, respectively. Of the 117 patients with IAC, 43 (36.8%) had focal IAC and 41 (34.5%) had diffuse IAC. In patients with diffuse IAC, 25 had multiple diffuse IAC. All diffuse IACs were found to be medial IAC. Risk factors (Table 1) of different patterns of IAC were shown and the identifications of confounding factors for FNO and END **(**Table S1) were shown in supplemental materials.

**Table 1:** Comparisons of baseline characteristics between different patterns of intracranial arterial calcification (IAC).

Characteristics	Intimal IAC			Medial IAC			Diffuse/focal IAC		
	Absent (*n* = 33)	Present (*n* = 97)	*p*‐value	Absent (*n* = 46)	Present (*n* = 84)	*p*‐value	Focal IAC (*n* = 76)	Diffuse IAC (*n* = 41)	*p*‐value
Male sex, *n* (%)	22 (66.7)	59 (60.8)	0.550	32 (69.6)	49 (58.3)	0.206	48 (63.2)	24 (58.5)	0.624
Age, mean ± SD	60.67 ± 16.89	65.97 ± 12.22	0.054	54.26 ± 12.00	70.30 ± 10.99	<0.001	62.36 ± 11.75	74.12 ± 9.51	0.001
Smoking, *n* (%)	11 (33.3)	36 (37.1)	0.696	27 (58.7)	20 (23.8)	<0.001	31 (40.8)	10 (24.4)	0.076
Drinking, *n* (%)	11 (33.3)	27 (28.8)	0.549	18 (39.1)	20 (23.8)	0.066	26 (34.2)	9 (22.0)	0.167
Diabetes, *n* (%)	6 (18.2)	30 (30.9)	0.158	10 (21.7)	26 (31.0)	0.262	22 (28.9)	13 (31.7)	0.756
Hypertension, *n* (%)	16 (48.5)	62 (63.9)	0.118	18 (39.1)	60 (71.4)	<0.001	46 (60.5)	30 (73.2)	0.171
Hyperlipidemia, *n* (%)	4 (12.1)	13 (13.4)	1.00	5 (10.9)	12 (14.3)	0.581	14 (18.4)	2 (4.9)	0.042
Atrial fibrillation, *n* (%)	8 (24.2)	13 (13.4)	0.144	4 (8.7)	17 (20.2)	0.087	9 (11.8)	11 (26.8)	0.040
History of stroke or TIA, *n* (%)	9 (27.3)	34 (35.1)	0.412	9 (19.6)	34 (40.5)	0.015	24 (31.6)	17 (41.5)	0.285
History of ischemic heart disease, *n* (%)	7 (21.2)	16 (16.5)	0.540	4 (8.7)	19 (22.6)	0.047	11 (14.5)	11 (26.8)	0.103

SD, standard deviation; TIA, transient ischemic attack.

### IAC and baseline NIHSS

No significant correlation was found by independent t‐tests between baseline NIHSS and the presence of intimal IAC or medial IAC. In patients with IAC, baseline NIHSS was significantly higher in patients with diffuse IAC than in patients with focal IAC (9.54 ± 8.21 vs. 5.67 ± 4.79, *p* = 0.011) (Table 2). Patients with multiple diffuse IAC had even higher baseline NIHSS compared with patients with focal or single diffuse IAC (12.04 ± 8.76 vs. 5.66 ± 4.94, *p* = 0.002). After adjusting for confounding factors, multivariable linear regression suggested that multiple diffuse IAC was correlated with higher baseline NIHSS (B coefficient 4.554; 95% CI, 1.859–7.250, *p* < 0.001).

**Table 2:** Comparison of the baseline National Institute of Health Stroke Scale (NIHSS) between different patterns of intracranial arterial calcification (IAC).

IAC	Baseline NIHSS, mean ± SD	*p*‐value
Intimal IAC		
Presence	6.55 ± 6.45	0.182
Absence	8.33 ± 7.07	
Medial IAC		
Presence	7.56 ± 6.92	0.195
Absence	5.98 ± 6.01	
Diffuse IAC	9.54 ± 8.21	0.011^a^
Focal IAC	5.67 ± 4.79	
Multiple diffuse IAC	12.04 ± 8.76	0.002^a^
Focal or single diffuse IAC	5.66 ± 4.94	

SD, standard deviation.

^a^ *p* < 0.05.

### IAC and clinical outcome

For the 130 patients, 118 received IVT only and 12 patients underwent EVT bridging to IVT. After reperfusion therapy, 83 patients (63.8%) had FNO. The presence of medial IAC or intimal IAC was not significantly associated with FNO. In patients with IAC, diffuse IAC was found to be associated with FNO (*p* = 0.047). Similar finding was identified in multiple diffuse IAC, compared with focal or single diffuse IAC (*p* = 0.024) (Table 3). After adjusting for confounding factors (Table 4), multivariable binary logistic regression suggested that multiple diffuse IAC was independently associated with FNO (adjusted OR 0.362; 95% CI, 0.146–0.893). Among the patients who received EVT, four had diffuse IAC and eight had focal IAC. The occurrence of FNO was not observed after EVT in the 4 patients who had diffuse IAC. After excluding the 12 patients receiving IVT plus EVT, multivariate binary logistic regression demonstrated that multiple diffuse IAC was correlated with less frequent FNO (adjusted OR, 0.398; 95% CI, 0.161–0.979). During the first 10 days after reperfusion therapy, 22 patients (16.9%) experienced END. Intimal IAC, medial IAC, or diffuse IAC was not found to be correlated with the occurrence of END (Table 3).

**Table 3:** Comparison of favorable neurologic outcome (FNO) and early neurological deterioration (END) between different patterns of intracranial arterial calcification (IAC).

IAC	FNO			END		
	Yes (*n* = 83)	No (*n* = 47)	*p‐*value	Yes (*n* = 22)	No (*n* = 108)	*p*‐value
Intimal IAC						
Presence, *n* (%)	63 (75.9)	34 (72.3)	0.654	16 (72.7)	79 (73.1)	0.968
Medial IAC						
Presence, *n* (%)	49 (59.0)	35 (74.5)	0.077	15 (68.2)	69 (63.9)	0.701
**Patients with IAC**	**Yes (*n* = 74)**	**No (*n* = 43)**		**Yes (*n* = 20)**	**No (*n* = 97)**	
Diffuse IAC, *n* (%)	21 (28.4)	20 (46.5)	0.047^a^	8 (40.0)	33 (34.0)	0.610
Multiple diffuse IAC, *n* (%)	11 (14.9)	14 (32.6)	0.024^a^	4 (20.0)	21 (21.6)	1.00

SD, standard deviation.

^a^ *p* < 0.05.

**Table 4:** Multivariable binary logistic regression on intracranial arterial calcification (IAC) and favorable neurologic outcome (FNO).

	Favorable neurologic outcome	*p*‐value
	Odds ratio (95% confidence interval)	
Crude association		
Diffuse IAC	0.456 (0.208–0.998)	0.049^a^
Multiple diffuse IAC	0.362 (0.146–0.893)	0.027^a^
Model‐1: Adjusted for confounding factors (atrial fibrillation and history of ischemic heart disease)		
Diffuse IAC	0.519 (0.232–1.163)	0.111
Multiple diffuse IAC	0.362 (0.146–0.893)	0.027^a^
Model‐2: Adjusted for confounding factors (age, atrial fibrillation, and history of ischemic heart disease)		
Diffuse IAC	0.615 (0.253–1.491)	0.282
Multiple diffuse IAC	0.362 (0.146–0.893)	0.027^a^

^a^ *p* < 0.05.

The inter‐rater reliabilities of IAC presence, IAC pattern (intimal and medial), and IAC involvement (focal and diffuse) were evaluated separately. The weighted kappa of IAC presence was 0.904 (95% CI, 0.839–0.969, *p* < 0.001), the weighted kappa of IAC pattern (intimal and medial) was 0.877 (95% CI, 0.760–0.993, *p* < 0.001), and the weighted kappa of IAC involvement (diffuse and focal) was 0.871 (95% CI, 0.747–0.995, *p* < 0.001).

## Discussion

The present study demonstrated that compared with focal IAC, the diffuse pattern of IAC was correlated with higher baseline NIHSS, less favorable neurologic outcome in the acute stage of ischemic stroke after reperfusion therapy. Considering all diffuse IACs were found medial IAC, it suggested that a larger involvement of medial IAC may be associated with less favorable therapeutic effect of reperfusion therapy during the acute stage of ischemic stroke.

We identified that patients with a diffuse pattern of medial IAC had higher baseline NIHSS compared with patients focal IACs, suggesting a correlation between medial IAC and severe neurologic dysfunction. This may account for a larger infarct volume in 5 to 7 days after stroke onset reported in patients with medial IAC than those with intimal IAC.^10^ Severe neurologic dysfunction may be due to arterial stiffening caused by medial IAC^18^ which could result in impaired vascular compliance of the distal vessels.^18^, ^19^ A state of distal hypoperfusion might arise from the restriction of vessels, therefore leading to microvascular failure.^20^, ^21^ In fact, it has been reported that IAC may be correlated with imaging markers of small vessel disease, including white matter hyperintensities, lacunes, and cerebral microbleeds.^22^, ^23^, ^24^ In patients with small vessel occlusion, those had heavier IAC density were demonstrated to have higher risk of stroke recurrence.^25^ These suggested the latent connection between IAC and small vessel disorders.

This study firstly identified that a diffuse pattern of medial IAC was associated with less favorable neurologic outcome after reperfusion therapy in the acute stage, which was partly different from prior studies suggesting that patients with medial IAC might benefit from EVT^10^ or IVT^26^ in functional outcome. In fact, prior studies did not evaluate the involvement of IAC, compared with our present study. Intimal IAC commonly coexists with atherosclerosis that is known to cause endothelial damage.^7^, ^8^, ^27^, ^28^, ^29^ As a consequence, the presence of intimal IAC might suggest a proneness of endothelial damage that is vulnerable to secondary injury after reperfusion. Compared with intimal IAC, medial IAC was reported in a previous study to be associated with better collateral status in patients who received IVT.^26^ This may correlate to the association between medial IAC and better functional recovery in previous studies, as good collateral filling may predict successful reperfusion,^30^ favorable outcome, and less recurrent strokes.^31^, ^32^ However, opposite findings were also reported in studies which included patients with EVT,^10^, ^33^ suggesting poorer collateral circulation in patients with medial IAC. We hypothesize that the discrepancy might result from the unstratified involvement of medial IAC that account for potential vascular injuries. In contrast to intimal IAC that is related to large artery disease,^7^, ^8^, ^34^ medial IAC might be associated with cerebral small vessel disease. When a large intracranial artery is occluded, the blood supply tends to be more dependent on the capacity of microvascular beds than that in branch occlusion. Considering the function of small vessel be hypothetically interfered by vasodilation restriction due to elevated arterial stiffness, a poorer collateral status might be observed among patients with severe medial IAC in studies based on EVT. This could also explain the findings in our study: When medial IAC emerges initially as a focal lesion, it may not have the capacity to affect the overall cerebral vascular bed. As medial IAC evolves and turns into a diffuse pattern, the status of microvascular beds might be more significantly damaged, thus leading to worse neurologic outcome in ischemic stroke.

One considerable novelty of this study is that we further categorized the patterns of IAC based on its degree of involvement in intracranial arteries. Calcification with long trajectory along the intracranial arteries is factually a common but unstudied phenomenon in clinical practice. It is notable that prior studies suggested that “severe” (larger volume, thickness or circumference) calcification in the intracranial ICA was related to incomplete arterial revascularization^3^ and prolonged procedure time^35^ in patients who had EVT. Since those two studies did not classify IAC pattern,^3^, ^35^ it is conceivable that arterial stiffening led by severe medial IAC may play an important role in affecting the outcome of EVT. By identifying severer neurologic dysfunction in patients with diffuse medial IAC than in focal medial IAC, we could hypothesize that diffuse medial IAC is a notable biomarker of the severe global medial calcification in cerebral vessel beds. Apart from the possible damage of global medial IAC on microvascular beds, local vascular compliance may also influence the outcome of EVT. With a long trajectory of medial IAC, the compliance of the vessels may be limited, leading to abnormal vessel compliance or impeded pathway of the instruments led by extensive IAC. According to our results as well as other similar studies, we may speculate that severe arterial calcification, such as diffuse medial calcification, could lead to poor clinical outcome due to relatively low successful rate of thrombectomies. Due to the small number of patients receiving EVT plus IVT therapy in our study, we need to test our speculations by recruiting more stroke patients in our future study. Another novelty of the present study is the analysis on mixed IAC pattern. We found that mixed IAC pattern may predict better functional recovery for stroke patients, suggesting a possibility of benefit from reperfusion therapy in patients with complex vascular status (both atherosclerosis and arterial stiffening), which may be inconsistent with general consensus. These current findings in our study may provide new insights into risk stratification and clinical managements in ischemic stroke.

Arterial calcification is a process of osteochondrogenic transformation. During the formation of calcification, the architecture of the vascular wall may be distorted,^36^ thereby causing vascular change, such as cerebral microbleeds.^22^ Stroke patients with larger IAC volume were found more prone to have hemorrhagic transformation after IVT^4^ and the presence of IAC may be related to intracerebral hematoma expansion.^37^ Endothelial impairment led by IAC may account for the increased permeability of brain blood barrier,^38^ which leads to subsequent hemorrhagic events. Although we grouped cerebral hemorrhage with stroke progression and stroke recurrence, evidence from other studies might validate our findings, with no significant difference in the frequency of cerebral hemorrhage between different IAC patterns.^10^, ^26^ Another cohort‐based study also documented similar frequencies of overall brain hemorrhage between patients with different IAC patterns.^39^ In our study, IAC was categorized into pathological patterns while other studies focused on the volume or density. Future studies on the volume or density of specific IAC patterns might be needed for the correlation between IAC and hemorrhagic events.

This present study has several limitations. First, in the present study, whether the neurologic outcome in the acute stage was associated with intermediate prognosis was not evaluated. It was reported that IAC pattern may be related to 90‐day functional outcome. Investigating the correlation between early and intermediate clinical outcome and their correlations with diffuse IAC in future studies might provide further evidence for clinical practice, such as rehabilitations for disabled patients. Second, this is a cohort from single stroke center, in which bias may exist during inclusion and treatment. Third, compared with previous studies with thousands of subjects, the cohort in this study was relatively small. With larger cohort, more correlations might be revealed between IAC and cerebrovascular diseases in the future.

## Conclusion

This present cohort‐based prospective study identified a potential impact of IAC pattern on the acute clinical outcome of reperfusion therapy. Diffuse IAC is associated with severer neurologic dysfunction and less favorable neurologic outcome from reperfusion therapy, suggesting this pattern of medial IAC may be a biomarker for clinical assessment and therapeutic strategies. Our findings may enrich the current understanding the impact of IAC pattern and provide insights into future clinical managements on ischemic stroke.

## Author Contributions

Jianrong Zheng, Cong Liu, Yajing Cheng, and Jun Hu performed the material preparation and data collection. Heng Du and Xuelong Li performed data analysis. Heng Du wrote the first draft of the manuscript. Heng Du conducted the manuscript revisions under the advice of Xiangyan Chen, Wenjie Yang, and Daniel Bos. All authors contributed to conception and design of this study and approved the final manuscript.

## Conflict of Interest

The authors declare no conflict of financial interests in this study.

## Supporting information


Table S1.
