# Supplementary material for: The correlation between intracranial arterial calcification and the outcome of reperfusion therapy
Source: Ann Clin Transl Neurol. 2023 Apr 23;10(6):974–82. doi: 10.1002/acn3.51780 (PMC10270261; doi:10.1002/acn3.51780)
Supplement: Supplementary file 1 — Table S1. [file ACN3-10-974-s001.docx]

**Table S1***.* Comparisons of vascular risk factors in favorable neurologic outcome (FNO) and in early neurological deterioration (END).

|  | FNO | | | | END | | | |
| --- | --- | --- | --- | --- | --- | --- | --- | --- |
|  | No (n=47) | Yes (n=83) | | *p* value | No (n=108) | | Yes (n=22) | *p* value |
| Male sex, n (%) | 26 (55.3) | 55 (66.3) | | 0.216 | 69 (63.9) | | 12 (54.5) | 0.410 |
| Age, mean ± SD | 68.60 ± 12.88 | 62.37 ± 13.69 | | 0.012 | 67.27 ± 11.85 | | 64.08 ± 14.01 | 0.321 |
| Smoking, n (%) | 15 (31.9) | 32 (38.6) | | 0.449 | 37 (34.3) | | 10 (45.5) | 0.319 |
| Drinking, n (%) | 11 (23.4) | 27 (32.5) | | 0.772 | 30 (27.8) | | 8 (36.4) | 0.420 |
| Diabetes, n (%) | 15 (31.9) | 21 (25.3) | | 0.418 | 29 (26.9) | | 7 (31.8) | 0.635 |
| Hypertension, n (%) | 29 (61.7) | 49 (59.0) | | 0.766 | 64 (59.3) | | 14 (63.6) | 0.702 |
| Hyperlipidemia, n (%) | 7 (14.9) | 10 (12.0) | | 0.644 | 13 (12.0) | | 4 (18.2) | 0.666 |
| Atrial fibrillation, n (%) | 12 (25.5) | 9 (10.8) | | 0.029 | 16 (14.8) | | 5 (22.7) | 0.358 |
| History of stroke or TIA, n (%) | 15 (31.9) | 28 (33.7) | | 0.832 | 36 (33.3) | | 7 (31.8) | 0.890 |
| History of ischemic heart disease, n (%) | 12 (25.5) | 11 (13.3) | | 0.078 | 19 (17.6) | | 4 (18.2) | 1.000 |
| Stroke territory |  |  | |  |  | |  |  |
| Anterior circulation stroke, n (%) | 39 (83.0) | 59 (71.1) | | 0.130 | 77 (71.3) | | 21 (95.5) | 0.017 |
| Posterior circulation stroke, n (%) | 8 (17.0) | 24 (28.9) | |  | 31 (28.7) | | 1 (4.5) |  |
| ASPECTS score |  |  | |  |  | |  |  |
| ASPECTS, mean ± SD | 9.46 ± 0.72 | | 9.61 ± 0.79 | 0.140 | 9.57 ± 0.70 | 9.48 ± 0.98 | | 0.878 |
| pc-ASPECTS, mean ± SD | 9.50 ± 0.76 | | 9.54 ± 0.93 | 0.654 | 9.53 ± 0.88 | 9.00 ± 0.00 | | 0.438 |

SD, standard deviation; TIA, transient ischemic attack; ASPECTS, The Alberta Stroke Program Early CT score; pc-ASPECTS, Posterior Circulation ASPECTS.
